# Baclofen approval in france: A balance between two conceptions of medicine

**DOI:** 10.1192/j.eurpsy.2021.101

**Published:** 2021-08-13

**Authors:** B. Rolland

**Affiliations:** Addiction Medicine, Université Lyon 1, CHU de Lyon, CH Le Vinatier, Lyon, France

**Keywords:** baclofen, alcohol use disorder, drug labeling, pharmacotherapy

## Abstract

In October 2018, France became the first country to officially approve baclofen for alcohol use disorder (AUD), even if the French Drug Agency (ANSM) officially stated that the efficacy of baclofen in AUD could be not established at this stage, in the light of the available evidence. The decision of the ANSM comprised obvious political aspects, as baclofen approval followed a decade-long practice of off-label prescription, where doses used could reach 300 mg per day or more. This situation led to a prolonged and ferocious debate between those who questioned such a widespread and unevidenced practice, and those who defended the place of an “common sense” empirical medicine. The French story of baclofen echoes other similar controversial off-label prescribing practices in the country, from the pioneer use buprenorphine for opioid use disorder in the 1990s, to the more recent off-label use of hydroxychloroquine during the COVID-19 outbreak. In each case, similar “pros” and “cons” arguments were opposed, highlighting the difficult interpenetration between evidence-based medicine on the one hand, and on-the-ground practice on the other hand.

**Disclosure:**

Benjamin Rolland declare having received fees for lectures and expertise from Ethypharm. He was the principal investigator of a phase-1 study funded by Ethypharm

